# Elements of fatherhood involved in the gestational period: a scoping review

**DOI:** 10.1590/0034-7167-2023-0029

**Published:** 2024-05-03

**Authors:** Willyane de Andrade Alvarenga, Maria da Conceição Silva Castro Sousa, Joice Kelly Lima de Sales, Rhyquelle Rhibna Neris, Francine DeMontigny, Lucila Castanheira Nascimento

**Affiliations:** I Centro Universitário Santo Agostinho.Teresina, Piauí, Brazil; II Universidade de São Paulo. Ribeirão Preto, São Paulo, Brazil; III Université du Québec en Outaouais, Département des sciences infirmières. Gatineau, Canada

**Keywords:** Prenatal Care, Paternity, Fathers, Pregnancy, Review., Atención Prenatal, Paternidad, Padre, Embarazo, Revisión., Cuidado Pré-Natal, Paternidade, Pai, Gravidez, Revisão

## Abstract

**Objective:**

To identify in the literature and summarize the elements and characteristics of fatherhood involved during pregnancy.

**Method:**

Scoping review that used PRISMA-ScR guide to report this review. Searches were carried out in PubMed, CINAHL, PsycInfo, LILACS and Scopus. Google search engines and public health agency websites assisted in searches of gray literature and Rayyan in screening studies.

**Results:**

A total of 406 articles were identified, of which 16 made up the final sample. Five elements make up an involved fatherhood: feeling like a father, being a provider and protector, being a partner and participant in pregnancy, participating in prenatal appointments and feeling prepared to take care of a baby.

**Conclusion:**

Fathers want to be involved in prenatal care, but feel excluded from this process. Public policies that encourage paternal involvement and healthcare professional training to better welcome and promote paternal involvement are of paramount importance.

## RESUMO

**Objetivo:**

Identificar na literatura e sintetizar os elementos e as características da paternidade envolvida durante a gestação.

**Método:**

*Scoping review* que utilizou o guia PRISMA-ScR para reportar esta revisão. Buscas foram realizadas nas bases PubMed, CINAHL, PsycInfo, LILACS e Scopus. Os mecanismos de busca do Google e sites de órgãos de saúde pública auxiliaram nas buscas na literatura cinzenta e o software Rayyan na triagem dos estudos.

**Resultados:**

Foram capturados 406 artigos, dos quais 16 compuseram a amostra final. Cinco elementos compõem uma paternidade envolvida: sentir-se pai, ser provedor e protetor, ser parceiro e participativo na gravidez, participar das consultas de pré-natal e sentir-se preparado para cuidar do bebê.

**Conclusão:**

O pai deseja estar envolvido nos cuidados pré-natais, porém sente-se excluído deste processo. Políticas públicas que estimulem o envolvimento paterno e a capacitação de profissionais de saúde para melhor acolher e promover o envolvimento paterno são de suma importância.

## RESUMEN

**Objetivo:**

Identificar en la literatura y resumir los elementos y características de la paternidad involucrada durante el embarazo.

**Método:**

Scoping review que utilizó la guía PRISMA-ScR para informar esta revisión. Las búsquedas se realizaron en las bases de datos PubMed, CINAHL, PsycInfo, LILACS y Scopus. Los motores de búsqueda de Google y los sitios web de agencias de salud pública ayudaron en las búsquedas de literatura gris y el software Rayyan en estudios de detección.

**Resultados:**

Se identificaron 406 artículos, 16 de los cuales constituyeron la muestra final. Cinco elementos componen una paternidad involucrada: sentirse padre, ser proveedor y protector, ser pareja y partícipe del embarazo, participar en las consultas prenatales y sentirse preparado para cuidar a un bebé.

**Conclusión:**

Los padres quieren participar en la atención prenatal, pero se sienten excluidos de este proceso. Las políticas públicas que fomenten la participación paterna y la formación de profesionales de la salud para acoger y promover mejor la participación paterna son de suma importancia.

## INTRODUCTION

Pregnancy and childbirth are physiological events that involve physical and emotional changes in parents’ lives. During pregnancy, women and their partners need prenatal care to prevent possible complications during pregnancy and childbirth^(1)^. Men must be part of the entire pregnancy process, which should not be linked only to women^(2)^. Fathers’ participation fathers during this period is important so that they feel confident and safe to play their role even before childbirth, in addition to strengthening family and emotional ties^(3)^.

Paternal involvement in prenatal care provides support and comfort to women. Evidence shows that it can even minimize anxiety and reduce working time^(4)^. It is also an opportunity for them, together with their partners, to gain knowledge regarding baby care and also to take care of their own health^(5-6)^. Furthermore, men will be able to effectively experience each moment of fatherhood, making the birth stage safer, participatory, dignified and exciting, creating an even greater emotional bond with women and their children^(4)^.

During their partners’ pregnancy, men go through transitions until they reach fatherhood, as it is a period of family adaptation, in which fathers feel concerned and feel the need to provide important care for their children^(7)^. This concern can lead to experience couvade syndrome, which comes from the French and means “to cry”, and this can occur in response to feelings of anxiety and excessive concern about the woman and the baby^(8-9)^. Although it is clear that, right at the beginning of pregnancy, physical changes are more noticeable in women. Studies show that some men, throughout this transition, may experience some physiological and physical changes, such as weight gain, muscle pain, insomnia and extreme fatigue^(10)^. Men can create expectations, feel anxious and insecure, as this is the time when they prepare themselves emotionally and financially for the arrival of a new member in the family^(11)^.

Future fathers feel the need to take a more active role during their partners’ pregnancy^(10)^. There are multiple determinants related to paternal involvement, which include individual, family, extra-family and cultural aspects^(12)^. The understanding of fatherhood is complex and variable over time, focusing on social dimensions and based on multiple roles in addition to biological or procreative aspects, involving attitudes such as supporting the family financially, interacting directly with children in providing care and support mothers emotionally^(13)^. Challenges can be encountered in this process, such as post-childbirth care for mothers and babies, which make fathers feel excluded^(14)^. Interaction, availability and responsibility towards the child are defined as components of paternal involvement^(13)^, which may vary depending on the context and child’s age^(12)^.

The benefits of paternal involvement for children, mothers and fathers themselves are clear^(15)^. However, the challenges for men’s/fathers’ involvement in prenatal care involve sociodemographic, sociocultural and barriers related to health services^(16)^. Therefore, it is necessary to reiterate the importance of this support during the transition to fatherhood as well as studies that seek to understand paternal experiences and needs during their wives’ pregnancy and childbirth^(17)^. It is also necessary that, in health environments, men’s participation in prenatal and maternal health appointments is encouraged by professionals, which can make them reflect and glimpse their behavior as fathers^(18)^. Encouraging them to attend appointments can, even at the beginning of pregnancy, improve access to knowledge about reproduction and health care as well as their involvement in maternal and neonatal care^(19-20)^ and in decision-making related to care^(21)^.

Although the literature shows studies on fathers’ involvement during their partner’s pregnancy, none of these reviews have, to date, summarized the aspects related to this involvement. It is therefore essential to understand how it happens during pregnancy as well as the elements and characteristics that make up a fatherhood involved, as this information can assist health professionals in developing interventions to welcome, engage and promote paternal involvement during prenatal care. Scoping reviews are a useful form of evidence synthesis for nursing professionals and present opportunities for researchers to review a wide range of evidence^(22)^. Given this gap in knowledge, the present scope review was proposed.

## OBJECTIVE

To identify in the literature and summarize the elements and characteristics of fatherhood involved during pregnancy.

## METHODS

### Study design

This is a scoping review, which covered six stages: 1. Topic identification and research question selection (“What aspects are related to motherhood involved during the gestational period?”); 2. Identification of relevant studies; 3. Study selection; 4. Categorization of selected studies; 5. Analysis and interpretation of results; and 6. Presentation of knowledge review/synthesis^(23)^. The Preferred Reporting Items for Systematic Reviews and Meta-Analyzes extensions for Scoping Reviews (PRISMA-ScR) was used to guide and report the essential items of this review^(24)^.

### Search strategy and data sources

PCC tool^(25)^ (P: Population, C: Concept and C: Context) was adopted to develop the research question and search strategy. The searches were carried out independently by two reviewers, during the month of August 2020, using electronic databases: PubMed (National Library of Medicine); CINAHL (Cumulative Index to Nursing and Allied Health Literature); PsycInfo (American Psychological Association), LILACS (Latin American and Caribbean Literature in Health Sciences); and Scopus. These databases were chosen for their relevance and impact in bringing together research in health and nursing as well as for concentrating the largest number of abstracts and citations relevant to the focus of the study. The search was performed without a time limit. The final limit of the search date considered studies developed before the COVID-19 pandemic, since this context can change the phenomenon and requires an individual analysis. The study search process is described using the PRISMA flowchart^(26)^. The descriptors (MeSH, ENTREE, and DeCS) fathers, spouses, paternity, prenatal care, pregnancy and father-child relations) were used combined with keywords using Booleans AND and OR (Chart 1).

**Chart 1:** Search strategy used in PubMed, 2023

PCC		SEARCH TERMS (MeSH and keywords)
P (Population) Father/partner	#1	“Fathers”[Mesh] OR “Fathers” OR “Father” OR “Stepfather” OR “Stepfathers” OR “Step-father” OR “Step-fathers” OR “Spouses”[Mesh] OR “Spouse” OR “Husbands” OR “Husband”
C (Concep) Fatherhood	#2	“Paternity”[Mesh] OR “Fatherhood” OR “Fathering” OR “Paternity” OR “Paternities” OR “Paternal Role” OR “Prenatal Bonding” OR “Paternal Attitudes” OR “Father-Child Relations”[Mesh] OR “Father-Child Relations” OR “Father Child Relations” OR “Father-Child Relation” OR “Relation, Father-Child” OR “Relations, Father-Child” OR “Father-Child Relationship” OR “Father Child Relationship” OR “Father-Child Relationships” OR “Relationship, Father-Child” OR “Relationships, Father-Child”
C (Context) Pregnancy/prenatal care	#3	“Prenatal Care”[Mesh] OR “Prenatal Care” OR “Care, Prenatal” OR “Antenatal Care” OR “Care, Antenatal”
Results:	#1 AND #2 AND #3	

The search was not limited to peer-reviewed published literature. Google search engines and public health agency websites were used to find gray literature, which consists of manuals and clinical practice guidelines.

### Inclusion and exclusion criteria

The review included original articles, with a quantitative or qualitative approach, literature reviews, letters, editorials, abstracts and guidelines that focused on fathers’ involvement during pregnancy, as well as aspects or strategies to expand this involvement and that had fathers, or partners, as study participants. Articles published in Portuguese, English and Spanish were included. Theses and dissertations, articles that presented the results together with those of other participants (e.g., mothers and health professionals), developed with fathers under 18 years old and that focused on paternal involvement after a baby’s birth were also excluded.

### Data collection and organziation, and analysis of results

The studies found in the databases were exported to Rayyan QCRI (http://rayyan.qcri.org)^(27)^. Duplicate articles were removed, and titles and abstracts were screened based on pre-established inclusion and exclusion criteria, by two reviewers, independently. Conflicts between the two reviewers at this screening stage were resolved by a third reviewer. Pre-selected studies were organized in a Word spreadsheet and both reviewers independently read the articles in full to select the final sample. When there were disagreements about including an article, a third reviewer helped with resolution. Finally, data from studies related to authors, year of publication, objectives, methods, main results and implications were extracted by two reviewers using a specific form for scoping reviews containing fields to extract information. Such data were analyzed descriptively, validated by all authors, and then a table to characterize the studies was created. To answer the review question, codes were developed in a deductive and inductive manner, based on the results of studies, by three reviewers who jointly constructed a list of codes in an interactive process. The codes gave rise to themes that were discussed with all authors for agreement.

## RESULTS

**Figure 1:**
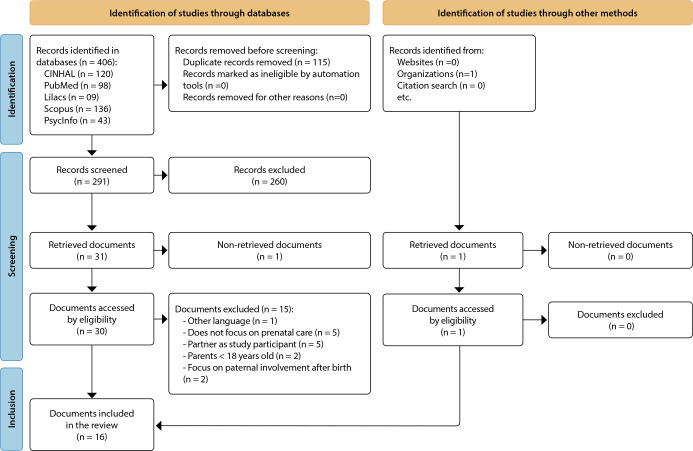
PRISMA^(26)^ Flowchart representing the literature review process

A total of 406 articles were identified through database searches. No studies were found by manual search in the reference list of included articles. A total of 115 duplicate studies were excluded, in a total of 291 unique articles, which had their titles and abstracts read independently by two reviewers. Based on the eligibility criteria, 260 studies were excluded, leaving 31 articles, which were read in full by two reviewers independently. Thus, 22 complete articles were excluded because they did not present original studies, were published in another language, did not focus on the prenatal period, had the partner as a study participant, presented fathers under 18 years old or focused on paternal involvement after birth. birth, in addition to the full study not found. In the end, the sample of this review is 16 studies (Figure 1).

### Characterization of included studies

Chart 2 illustrates the characteristics of included studies. They were developed in the United States (n=4)^(19,21,28-29)^, Australia (n=3)^(20,30-31)^, Brazil (n=2)^(32-33)^, Singapore (n=2)^(14,17)^, Sweden (n=1)^(34)^, Taiwan (n=1)^(35)^, England (n=1)^(36)^, Iran (n=1)^(37)^ and Jamaica (n=1)^(38)^.

**Chart 2:** Characterization of included studies (N=16)

First author, year and country	Objective/proposal	Study design	Characteristics of participants/included studies
Obrzut, 1976^(29)^ United States	Determine how expectant fathers define fatherhood, how they prepare for this moment and what their feelings about fatherhood are.	Qualitative study	N=20 first-time fathers. Ages from 20 to 40 years old, with an average of 27 years old. Gestational age in the last two months. All fathers were employees. Most were middle class. Thirteen pregnancies were planned.
Kao, 2004^(35)^ Taiwan	Explore the experience of expectant fathers during their wives’ third trimester of pregnancy.	Qualitative study	N=14 first-time fathers. Partners’ gestational age between 34-36 weeks.
Deave, 2008^(36)^ England	Explore first-time fathers’ needs in relation to care, support and education provided by health professionals during prenatal care, especially in relation to preparation for the transition to fatherhood and parenting skills.	Qualitative study	N=20 fathers from different backgrounds who were about to become fathers for the first time. N=18 fathers were interviewed again between 3 and 4 months postnatally. N=1 had moved. N=1 was unavailable for the second interview. Age varies between 19 and 37 years old. N=18 were White British. N=1 Asian. N=1 Brazilian. Variety of socioeconomic backgrounds, employment status varying from manual labor (n=7) to professional (n=6). N=1 was unemployed. N=1 receiving state disability benefit.
Poh, 2014^(17)^ Singapore	Provide an overview of evidence on fathers’ experiences and needs during their partners’ pregnancy and childbirth to identify any gaps in existing literature and practice.	Integrative review	25 studies, 6 quantitative and 19 qualitative. Eight studies reported fathers’ experiences during pregnancy, 13 during childbirth and 4 in both periods.
Tehrani, 2015^(37)^ Iran	Define fathers’ experience in their wives’ first pregnancy in a qualitative way and in the real environment scenario.	Qualitative study	N=26 fathers. Average age was 29 years, 55% of them were highly educated, 14% had higher education. 86% of fathers worked in the service sector, 9% were manual workers and 5% were office workers. Gestational age of the partner between the 32^nd^ and 40^th^ week.
Jeffery, 2015^(30)^ Australia	Assess fathers’ engagement levels in an Australian setting and determine whether the potentially modifiable factor of appointment by antenatal care providers influences fathers’ engagement.	Mixed method study	Average age of fathers was 31.3 years. Families with religious beliefs. Of the sample, 74% had a partner in the third trimester of pregnancy and 43% were first-time fathers.
Davis, 2015^(38)^ Jamaica	Discuss the role of fathers in pregnancy in Jamaica.	Discussion article	Not applicable.
Aguiar, 2015^(19)^ United States	Synthesize current literature regarding the effect of male prenatal care on non-HIV-related perinatal health outcomes in developing countries.	Systematic review	Seven studies were included in the review, all quantitative, longitudinal, prospective or comparative. Included studies originated from Asia and sub-Saharan Africa.
Johnsen, 2017^(34)^ Sweden	Illuminate first-time expectant fathers’ experience of participation during pregnancy in three Nordic countries.	Qualitative study	N= 31 fathers who lived in Sweden (Boras n=8, Hund n= 10), Denmark (Copenhagen n=8) and Finland (Helsinki n=5). Fathers recruited when the partner was 30 weeks pregnant or beyond. Ages: 24 to 43 years old. N=3 fathers were studying. N= 7 fathers had a university degree. N=21 fathers had a degree. N=0 unemployed fathers.
Ministry of Health, 2018^(33)^ Brazil	Present partners’ prenatal strategy.	Guide for healthcare professional	Not applicable.
Deibel, 2018^(28)^ United States	Assess the feasibility and acceptability of adding a 2-hour session for fathers (male partners only) within a prenatal care model group known as Centering Pregnancy (CP).	Pilot study	N=5 fathers already enrolled in the prenatal program, who had completed up to the eighth session of the appointment, fluent in English. All were married and lived with their partners. N= 3 fathers were having their first child. N=2 fathers were having their second child. Ages varied between 18 and 24 years old (n=1), 25 and 29 years old (n=2) and 30 and 35 years old (n=2). N=1 father identified as African American. N=3 fathers identified as white. N= 1 father refused to respond. N= 3 fathers had secondary education or higher. N= 1 father had a doctoral degree. N= 1 father did not answer.
Nash, 2018^(31)^ Australia	Examine how first-time fathers in rural Tasmania experienced fathers-only antenatal education/support groups.	Qualitative study	N=25 fathers living in three rural areas of Tasmania (South, Central Coast and North Midlands). They were ≥18 years old and about to become first-time fathers with a partner of at least 20 weeks’ gestational age. Fathers were between 24 and 43 years old. The majority lived in the countryside and self-identified as Anglo-Australian, and 50% had higher education.
Xue, 2018^(14)^ Singapore	Provide a literature overview on paternal involvement during the pregnancy and childbirth periods and the factors that influence this involvement.	Integrative review	A total of 31 studies were included, 17 quantitative, 9 qualitative and 5 reviews.
Tokhi, 2018^(20)^ Australia	Determine the effect of interventions to engage men during pregnancy, childbirth and childhood on mortality and morbidity.	Systematic review	Thirteen studies from nine countries were included in the review, three of which had experimental designs and ten were observational. Eight studies were conducted in South Asia, 3 in Southern or East Africa, 1 in Indonesia, and another in Turkey.
Cheng, 2019^(21)^ United States	Conduct a narrative literature review to explore fathers’ preferences, perspectives and involvement in perinatal decision-making.	Narrative review	A total of 13 studies were included in the review, the majority from Scandinavia. Twelve studies were qualitative in nature and 1 was quantitative. The primary studies included gestational and post-childbirth fathers, first-time fathers, and second-time fathers.
Bonifácio, 2020^(32)^ Brazil	Assess the PRENACEL Program implementation, a technological health communication resource, via SMS, for partners of pregnant women, as a strategy to improve men’s participation and involvement in prenatal care and childbirth.	Randomized controlled clinical trial	Average age of fathers was 30 years, with minimum 19 and maximum 54 years. N=186 fathers. N=62 from the PRENACEL group (who received the messages). N=73 from the non-PRENACEL group (partners who did not receive the messages, but belonged to the intervention Basic Health Units). N=51 from the control group. 51% declared themselves mixed race, 90% were working and 62% already had children.

The methodologies used were qualitative (n=6)^(29,31,34-37)^, systematic review (n=2)^(19-20)^, integrative review (n=2)^(14,17)^, randomized clinical study (n=1)^(32)^, narrative review (n=1)^(21)^, mixed method study (n=1)^(30)^, discussion study (n= 1)^(38)^, Ministry of Health manual (n=1)^(33)^ and pilot study (n=1)^(28)^.

### Elements of fatherhood involved in the gestational period

**Figure 2:**
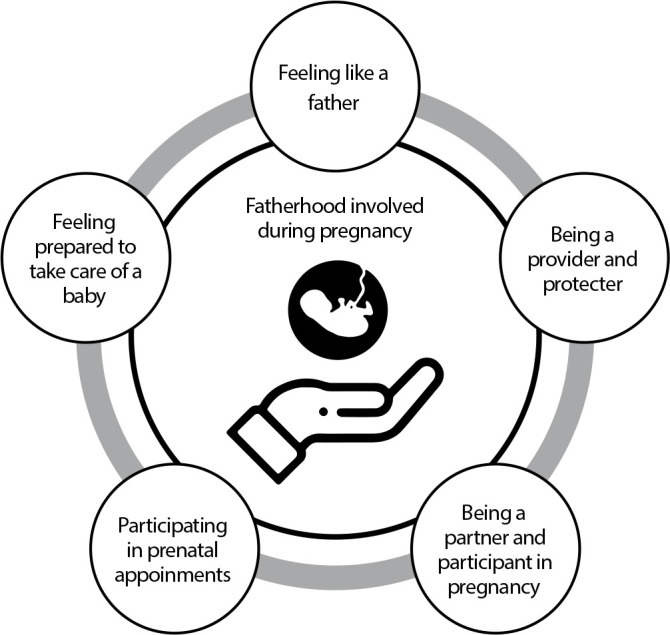
Elements of fatherhood involved in the gestational period

From the synthesis of the included studies, it was found that five elements make up a fatherhood involved during the gestational period: feeling like a father, being a provider and protector, being a partner and participant in pregnancy, participating in prenatal appointments and feeling prepared to take care of a baby (Figure 2).

#### Feeling like a father

Some fathers believe that the feeling of fatherhood for men begins with marriage, while for others it begins when their wives are pregnant^(17)^, or as soon as their children are born^(37)^. Studies show that fathers feel the presence of their children before birth, and this feeling encourages them to develop emotional dependence and a feeling of connection with their children^(17)^, enjoying coming home and spending 24 hours with their wives and children^(37)^. However, others do not identify any feeling of emotional dependence on their children^(37)^.

Fathers experience feelings of happiness, responsibility, the need for planning and pride with the news of the pregnancy. Following their cultural and religious beliefs, the majority were satisfied with their wives’ pregnancies and saw their children as a divine gift, being grateful to God^(37)^. Fear is also usually present at this stage, and only decreases when they see their wives’ abdomens growing^(21)^. There is also a feeling of need for fatherhood, as they believe that all men want someone to call them father^(34,37)^.

During the third trimester of pregnancy, paternal reports of a mix of emotions are common due to the proximity of their babies’ birth^(35)^. Although receiving information was an effective strategy to reduce insecurity, they experience feelings of anxiety and concern about their children’s and wives’ health^(17,21,34,37)^. Fathers also feel anxious about childbirth support, financial stability, breastfeeding assistance, and ways to soothe a crying baby^(17,21,28,37)^.

#### Being a provider and protector

The news of pregnancy initiates mental, psychological, social and physical changes in the fathers’ lives, which, together, contribute to paternal identity formation^(37)^. A maturation process begins as a partner and father, in order to become a role model for children and ensure a stable and economically viable home environment^(32,34)^. Given the awareness of the need to prepare themselves and the environment for the baby’s arrival, fathers take an active role during pregnancy. Their main role in the family is to be providers^(17,29)^, nurturers, caregivers^(17,29)^ and educators of children^(37)^.

For them, fathers are responsible for the family’s financial matters and mothers’ role is more important than theirs, as children are mothers’ duties^(37)^. Other studies show that they perform household chores and feel a sense of responsibility for ensuring that their partners adopt a healthy lifestyle during pregnancy^(29,34,37)^.

A baby’s arrival is experienced as a significant milestone, as it signifies the transition to fatherhood^(34-35,37)^. The feeling of fatherhood makes fathers be involved and feel prepared, emotionally and physically, to provide support and protection to children and partners^(34,37)^. Participants in one of the studies included in this review reported motivation to develop their personal qualities, as they wanted to serve as role models for their children^(36)^.

#### Being a partner and participant in pregnancy

Studies show that fathers begin to accept the reality of pregnancy throughout the first and second trimester, becoming more attentive to their wives and children and beginning to want to participate and be included in all aspects of pregnancy^(34)^. For them, fatherhood is humanized through greater participation in child rearing. Building child’s future is a value that must be passed from generation to generation and given to children: “Once you receive emotions and a sense of responsibility from your father, you are obliged to keep them within yourself and transfer them for the future generation, made up of their children”^(37)^.

Some choose not to share concerns with their partners, to protect them, and feel feelings of helplessness because they cannot alleviate their partner’s physical and emotional suffering^(34)^. Fathers realize that during the pregnancy period, wives become very sensitive and that being attentive is a way of expressing love to their wife and child^(37)^. Furthermore, promoting a sense of security for the pregnant partner, through support and protection, facilitated the joint transition to parenthood^(34-35,37)^.

In addition to viewing the baby during the ultrasound, fathers also document the progress of the pregnancy by taking photos of their partners^(17,34)^. Palpating the woman’s belly and feeling the child was perceived as a way of showing affection and establishing a bond with the baby and the partner, as, for them, being next to the “belly” is a way of remaining included in the process and participate in pregnancy^(34,37)^. For them, a very important moment was, before going to sleep, being able to touch their partners’ belly a little and talk to the baby, as it meant that they were doing something together^(34)^. When the baby reacted to the fathers’ voice, it created a feeling of being part of the pregnancy and they felt more connected^(14,17,34)^. Hearing the heartbeat and seeing the fetus through ultrasound examination encouraged them to continue participating in prenatal activities^(14)^.

Fathers recognize that a good partnership depends on mutual contributions to the couple’s relationship. Sharing the same values and having time to talk to their partner was an important part of the journey towards fatherhood^(34,36)^. Pregnancy improved the marital relationship^(17)^. Fathers adopted a series of strategies to compensate for the lack of involvement, such as talking to their wives about biological changes and attending prenatal exams^(34)^.

#### Participating in prenatal appointments

Fathers generally describe that attending prenatal appointments is important for the transition from fatherhood and understanding the evolution of pregnancy, as it is possible to see the baby’s position inside the uterus, estimate fetal growth and hear the fetal heartbeat, the which, for them, is considered real proof of pregnancy^(34)^. This experience promotes reflections about the baby and about how to create a family^(34)^. Furthermore, asking questions during the prenatal appointment is a way of demonstrating responsibility for the baby^(34)^.

However, for some, being present and accompanying their partners at prenatal appointments is difficult due to work or their employers’ lack of permission to take time off^(34,38)^. They report a feeling of isolation due to work and difficulty balancing personal and professional life^(36)^, as the following excerpt illustrates: “It’s my first time in the hospital and the baby is almost here. I feel unprepared and anxious, not participating due to work. I’m not ready at all”^(30)^. Furthermore, they feel pressured to attend prenatal exams, as men are expected to have the same obligations as women^(34)^.

There are also reports of a feeling of discouragement, due to the fact that some health professionals only approach women during appointment^(21,34)^. Others report feelings of exclusion in prenatal care, as care is more focused on women and only minimal effort appears to be made to meet paternal needs^(14)^. These fathers suggest that prenatal appointments be planned to include them and help them with the arrival of their first child^(17,36)^. When health professionals use the plural form when addressing the couple, fathers feel included^(34)^. Furthermore, paternal involvement in prenatal appointments can have a positive effect on their own health, as they take advantage of their presence in health units for activities related to pregnancy to take care of their own health^(33)^.

A study concluded that adding a fatherhood session for men only in prenatal care made fathers feel more involved in pregnancy and confident in providing support to their partner during pregnancy, as they were able to express feelings and thoughts and exchange experiences^(28)^. In another study, using technological communication resource via SMS allowed them to participate more in prenatal appointments and be more present at birth^(32)^.

#### Feeling prepared to take care of a baby

Although men feel excited about the idea of having a baby, they often express the feeling of always being watched as well as a lack of preparation and apprehension regarding the practical and general aspects of caring for a baby^(14,21,36)^. They want to be heard, both prenatally and postnatally, talking about their experiences of dealing with a new baby and obtaining information related to this role and the practices of caring for a baby^(14,17,21,36)^.

Prenatal classes, from the beginning of pregnancy, and group meetings, in addition to helping fathers understand paternal roles, enable them to feel more supported^(31,38)^. Carrying out educational activities with fathers is an effective intervention and increases the proportion of those who accompany their partners in prenatal^(20)^ and postnatal appointments^(19)^. In this sense, primary care is a suitable field for developing these educational activities^(33)^. Nurses, in prenatal appointments, must assess how a child’s arrival affects the entire family, encourage fathers to accompany their wives in prenatal appointments, welcome them and integrate them into the process^(29,38)^. The entire appointment is an opportunity to listen and create a bond between fathers and health professionals, providing clarification of doubts and guidance on relevant topics^(33)^.

Interventions that promote fathers’ involvement during prenatal care increase the demand for care, favoring more equitable decision-making by the couple in favor of maternal and neonatal health^(20)^, enhancing couples’ communication about pregnancy care^(19)^. Fathers reported that, when they had any doubts about how to properly exercise fatherhood, they turned to the mothers, close friends or their parents^(17,28)^. Other studies have shown that they feel safer talking to their mothers or other female relatives^(36)^. Fatherhood was widely seen as an intimate topic and perhaps inappropriate to discuss with other men, as they do not feel comfortable talking about it with their own parents or male friends, for fear of judgement^(31)^.

## DISCUSSION

This review brought together scientific evidence on the elements of paternity involved during the gestational period. Feeling like a father, being a provider and protector, being a partner and participant in pregnancy, participating in prenatal appointments and feeling prepared to take care of a baby were the themes presented in this review.

Scientific evidence considered the presence of a partner during prenatal care, childbirth and postpartum to be positive^(19)^. This evidence is in agreement with this review, which showed mental, psychological, social and physical changes triggered by pregnancy for fathers to obtain or develop the requirements considered necessary to be a father, which results in conformation of their paternal identity.

The results also showed that fathers take an active role during their partner’s pregnancy, as they understand and are aware of the need to prepare themselves and the environment for a baby’s arrival. According to them, having their own child is a significant milestone in building their identity as a man, as it delimits the transition from maturity to fatherhood. The literature also shows that, although they mostly take on the role of financial provider, the role of caregiver and emotional supporter of their partners begins to have space and characterizes paternal involvement^(3)^.

The transition to fatherhood is a challenging period for many men due to social expectation of paternal involvement^(40)^. The results of this review showed that they express feelings of anxiety and concern about their child’s and wife’s health and also in relation to childbirth. A systematic review of stress in fathers during the perinatal period showed increased levels of anxiety, stress and risk of depression during the transition period to fatherhood^(41)^.

This review found that fathers, to promote a sense of security for their partner, look for ways to provide support and protection. Similar results were found in the literature, in which it is described that men care about their partners and children and, therefore, play a role that they believe is theirs: providing support and protection^(21)^. Other concerns described by the studies were related to childbirth, financial stability, breastfeeding and ways to calm a crying baby in the future.

The review also showed that some fathers feel unprepared for a baby’s arrival. In this regard, encouraging partners to attend prenatal appointments can be seen as the first step towards providing more confidence. When paternal prenatal programs are offered to fathers, they are willing to participate^(42)^. Including them in prenatal care is, therefore, a way of involving them in a positive way in their partners’ pregnancy process^(17)^.

The feeling of fatherhood makes them involved and feel prepared, emotionally and physically, to provide support and protection for their children and partners^(19)^. However, this same study showed that some choose not to share concerns with their partners to protect them and thus feel powerless and unable to alleviate their physical and emotional suffering. It is believed that there is recognition by fathers that a partnership depends on mutual contributions in a relationship. Sharing the same values and taking the time to talk to their partners is an important part of the journey towards fatherhood. Fathers must participate in the pregnancy process^(17)^.

In Brazil, the Prenatal and Birth Humanization Program (PHPN)^(39)^, offered by the Ministry of Health, was created with the aim of ensuring access and quality assistance during prenatal care, childbirth and birth so that partners are also included in these actions. This review showed that fathers consider it important to attend prenatal appointments, as they can see the position of the babies inside the uterus, in addition to estimating fetal growth and hearing fetal heartbeat, which, for them, is considered proof that pregnancy is real.

The literature provides important explanations about the benefits for the family of fathers’ participation in prenatal care, and one of them is the strengthening of the couple’s emotional relationship^(43)^. The benefits of greater paternal involvement, including after birth in child care, have been demonstrated in systematic reviews to be associated with improved family functioning^(44)^ and reduced behavioral disorders in children^(45)^.

The Pre-Natal and Postpartum Technical Manual published in 2005 by the Brazilian Ministry of Health^(46)^ recommends that fathers’ participation during pre-natal appointments be motivated through their inclusion in activities developed in groups^(47)^. From this perspective, evidence in the literature shows that the multidisciplinary health team, mainly nurses, must have knowledge on the subject, welcoming fathers so that they can feel safe for the birth of their babies. During prenatal care, health professionals must assess how a child’s birth affects the entire family and encourage fathers to accompany their wives in prenatal exams, convincing them of the importance of their role, exploring their thoughts and feelings related to fatherhood^(48)^.

Often, some challenges make it difficult for men to be involved in caring for babies and receiving information, despite wanting to participate in their wives’ pregnancy process, which makes them feel excluded^(14)^. The fathers included in the studies complained about lack of time and impediments due to work to participate in prenatal appointments. Other challenges were cited in another study with English fathers, such as a lack of support from health professionals, both because there is poor communication and because they feel ignored in maternity wards, where they are treated as visitors^(49)^.

Therefore, the difficulty of balancing professional and personal life and still finding time to accompany their partners became evident in this review, as, not infrequently, fathers do not obtain permission or time off from work and are still under pressure to be present at appointments. In this context, it is essential that the health professional team offers inclusion activities for men, making the opening hours of activities more flexible to facilitate access^(50)^. Studies show that men’s active participation in all aspects related to women’s health is extremely important.

### Study limitations

This review had some limitations that need to be acknowledged. Few studies have been carried out on paternal involvement during pregnancy and few guidelines have been identified. There is, therefore, limited capacity to attest to the effectiveness of interventions to promote paternal involvement.

### Contributions to nursing, health or public policy

This scoping review expands the understanding of fathers’ involvement in the gestational period by describing the different characteristics and strategies of paternal involvement. Fathers’ participation in child care is a critical area of study, due to the number of research with the male population and the need for father-centered interventions. The importance of more research with men on the experience and subjective aspects of involved and caring fatherhood during the gestational period as well as the assessment of interventions that promote paternal involvement are reinforced.

It is extremely important to include fathers in care during the pregnancy period. This study reiterates the importance of developing public policies that favor paternal involvement and health professional training to welcome and encourage them to participate in prenatal care. This review shows a fatherhood in prenatal care with elements more inclusive of responsibilities for providing care to children and partners and less strictly related to financial contributions. Given this, it is important to understand the best way to involve fathers in interventions that aim to improve the effectiveness of their parenting and extend it to coparenting. Some interventions can be described as including men in prenatal appointments, sharing information, and providing an overview of pregnancy to make them feel more included and involved. Creating support groups with men who consider their needs, with inclusion activities and flexible hours to combine with work, is a strategy to facilitate their participation. The intervention context can involve different spaces, such as primary care, hospitals, community and employer.

## CONCLUSIONS

This scoping review analyzed the elements of fatherhood involved during the gestational period, describing paternal characteristics, needs and experiences throughout this period. It was evident that fathers experience conflicting feelings, such as joy, anxiety and worry, and although they want to be involved in their wives’ pregnancy, they feel excluded and repressed during this process. Just inviting them to participate in prenatal appointments may not be enough to demonstrate their importance related to maternal care, with babies and with themselves, since other factors contribute or harm this involvement, such as feeling like a father, be a partner and participant in pregnancy and feel prepared to take care of a baby. Public policies that encourage paternal involvement and the training of healthcare professionals to better welcome and promote paternal involvement from the gestational period are of paramount importance.
